# The quantitative sensory testing is an efficient objective method for assessment of nerve injury

**DOI:** 10.1186/s40902-015-0013-5

**Published:** 2015-05-03

**Authors:** Young-Kyun Kim, Pil-Young Yun, Jong-Hwa Kim, Ji-Young Lee, Won Lee

**Affiliations:** 1 Department of Oral and Maxillofacial Surgery, Section of Dentistry, Seoul National University Bundang Hospital, Gyeonggi, South Korea; 2 Department of Dentistry, Uijeongbu St. Mary’s Hospital, College of Medicine, The Catholic University of Korea, 222 Banpo-daero, Seocho-gu, Seoul 137-701 Republic of Korea

**Keywords:** Nerve injury, Somatosensory Evoked Potentials, Quantitative Sensory Testing, Thermography

## Abstract

**Background:**

This study evaluated Somatosensory evoked potentials (SEP), Quantitative sensory testing (QST), and thermography as diagnostic methods for nerve injury.

**Methods:**

From 2006 through 2011, 17 patients (mean age: 50.1 years) from OOOO Hospital who sought care for altered sensation after dental implant treatment were identified. The mean time of objective assessment was 15.2 months after onset.

**Results:**

SEP of Inferior alveolar nerve(IAN) was 15.87 ± 0.87 ms on the normal side and 16.18 ± 0.73 ms on the abnormal side. There was delayed N20 latency on the abnormal side, but the difference was not statistically significant. In QST, the abnormal side showed significantly higher scores of the current perception threshold at 2 KHz, 250 Hz, and 5 Hz. The absolute temperature difference was 0.55°C without statistically significance.

**Conclusion:**

These results indicate that QST is valuable as an objective method for assessment of nerve injury.

## Background

Because of increases in implants, extractions, and orthognathic surgical procedures, damage to the inferior alveolar nerve, a branch of the trigeminal nerve, has recently not been uncommon. The main cause of inferior alveolar nerve damage is mandibular third molar extraction, which accounts for more than half of the incidence [1].

It has been reported that permanent damage occurs in 3.6% and temporary damage in 8% of cases of mandibular nerve damage associated with third molar extraction [2]. The other causes are local anesthetic injection, endodontic treatment, orthognathic surgery, and implant surgery. Nerve damage can have various causes, but the assessment of the degree of nerve damage can vary depending on the evaluation method used. The evaluation of paresthesia after nerve injury is important for treatment choice, follow-up, and prognostic assessment [3].

The responses of an ideal diagnostic test in sensory nerve damage are positive in the damaged nerve and negative in normal nerves, and the test should be able to distinguish the extent of nerve damage accurately.

The conventional noninvasive tests for diagnosis of traumatic damage of the sensory nerves include static light touch detection, brush direction discrimination, two-point discrimination, the pin pressure nociceptive discrimination test, and thermal discrimination. These, however, are subjective and have the disadvantage of depending on the sensory response of the patient [4]. Moreover, these tests cannot evaluate and quantify the damage objectively when the patient complains of numbness or paresthesia subjectively.

The Somatosensory Evoked Potentials (SEP) test is an electrical physiological test that detects the change in the electrical potential of the peripheral or central nervous system evoked by peripheral sensory nerve stimulation. It is a relatively objective way to examine the radiation path from the peripheral nerve to the thalamic through the Ia fiber [5]. The SEP test is noninvasive, highly objective, and extremely reliable, and can be used to investigate trigeminal sensory hypoesthesia. SEP data are directly collected from the patient’s electroencephalography derived from the cerebral cortex [6].

To assess and quantify nerve function objectively, Quantitative Sensory Testing (QST) has been studied. Current Perception Threshold (CPT) has the advantage of being less time-consuming, requiring 10 min per test area. It can quantify the function of large myelinated (Aβ, 2KHz), small myelinated (Aδ, 250Hz), and unmyelinated nerves (C, 5Hz) by a double-blind technique [7].

Thermography diagnoses nerve damage by comparing clinical symptoms and the difference in the temperature of the left and right sides of the body, visualizing the body surface temperature that results from the altered blood flow at the painful area or lesion by detecting the infrared radiation emitted from the body. This has the advantages of being a non-invasive and not requiring exposure of the patient to radiation [8].

Here we evaluate the usefulness of SEP, QST, and thermography for objective assessment of sensory nerve injury.

## Methods

### Participants

This study included 17 patients (male: 7; female: 10) who sought treatment for altered sensation of the lower lip and chin at OOOO Hospital after dental implant treatment at a local clinic from 2006 through 2011. Their mean age is 50.1 years. The altered sensation regions were on the right side in eight cases and on the left side in nine cases. In all patients, symptoms occurred after implant installation in the posterior area. The implants were removed in eight cases and installed with an upper prosthesis in nine cases. Information about the reverse turn of the implant or re-installation after removal was not available. Age ranged from 32 years to 69 years, with a mean of 50.1 years. The cause of mandibular nerve damage was implant placement. The mean time of objective assessment was 15.2 months (Table 1). The study was conducted after obtaining approval from the institutional review board (IRB) for clinical studies. (IRB No.: B-1111-139-104) We have followed the guidelines of the Helsinki Declaration in this investigation.

**Table 1:** **Detailed information of the patients**

**Case**	**Age**	**Sex**	**Affected side**	**Period**	**Implant**
1	49	M	Rt	32	M
2	49	F	Lt	16	M
3	51	F	Lt	16	R
4	55	M	Lt	17	M
5	50	M	Rt	19	M
6	54	M	Rt	12	M
7	52	F	Rt	2	R
8	52	F	Rt	13	R
9	56	F	Lt	9	R
10	69	F	Rt	7	M
11	32	F	Rt	12	M
12	50	F	Lt	2	M
13	49	F	Lt	73	M
14	45	M	Lt	12	R
15	37	F	Rt	7	R
16	50	M	Lt	5	R
17	51	M	Lt	5	R

Period: from implant placement time to assessment time (month)

Rt: right, Lt: left, M: maintained, R: removed

### Methods

#### SEP

**Figure 1:**
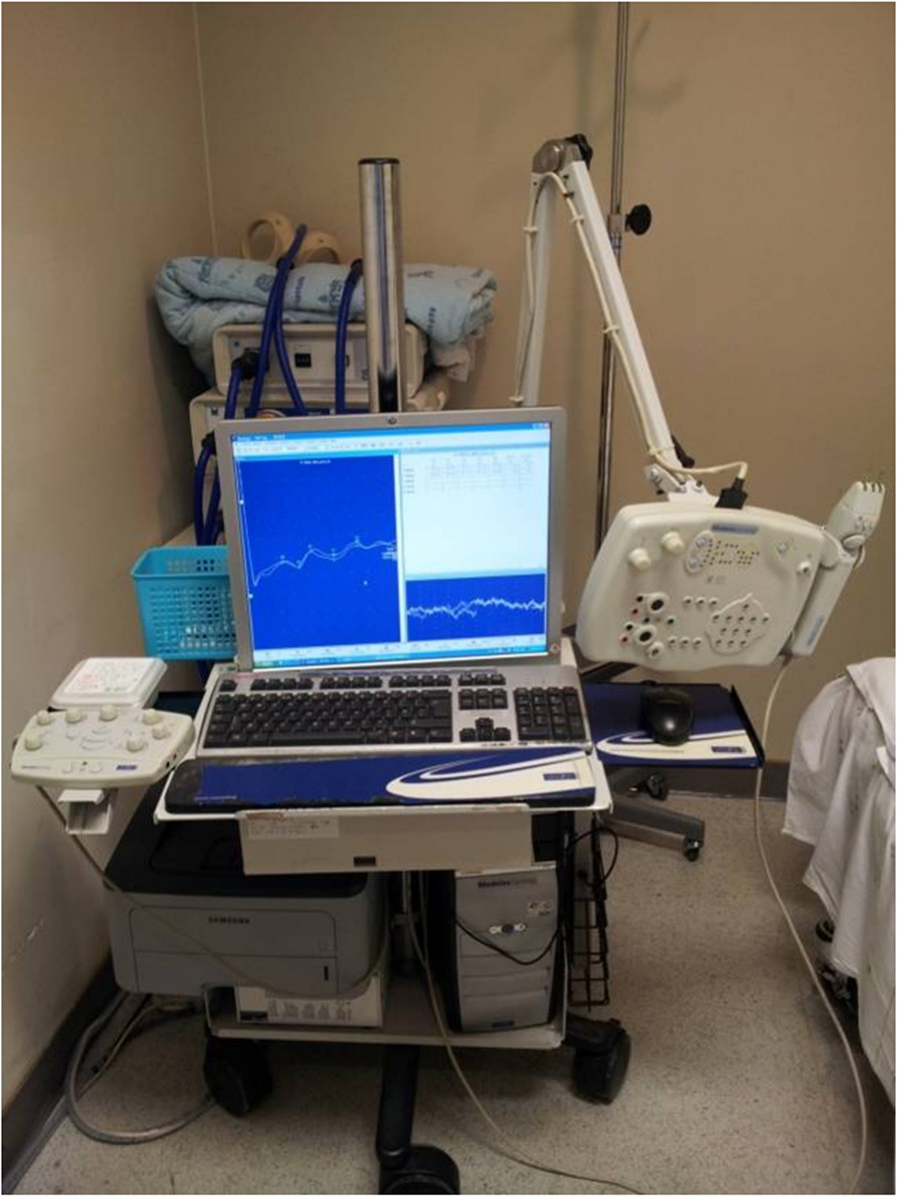
SEP equipment. Nicolet EDX-Synergy.

A short scalp electrode needle was attached while the patient is lying down. The electrode needle was placed on the skin to examine the area of interest. The abnormal pathway of the nerve from the head to hands or feet was examined using Nicolet EDX (CareFusion 209 Inc./Middleton, WI, USA). The patient should not move to get an accurate test result. The electrical stimulation was under 10 mA but it could be somewhat painful. It takes 30–60 minutes (Figure 1). The mean value of N20 latency (ms) of the abnormal and normal sides was compared for SEP.

#### QST

**Figure 2:**
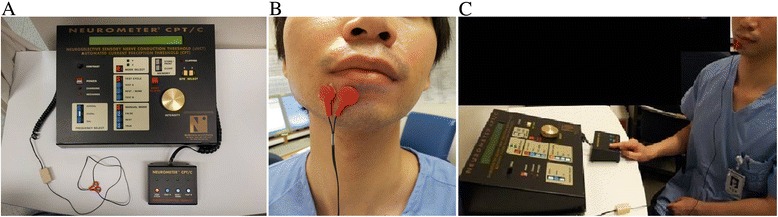
Methods of QST measurement. **A:** Neurometer CPT/C. **B:** Attach the electrode at the inspected area. **C:** The patient was asked to stop pressing the button when he or she detected minute electrical stimulation, vibration, pain, and/or heat from electrical stimulation using R-CPT (rapid-current perception threshold) mode.

The measurement site on the skin was wiped with an ethyl alcohol sponge, and a Goldtrode (Neurotron, Baltimore, MD, USA) bearing a thin layer of conductive gel was applied. The CPT after stimulation with three frequencies (2KHz, 250Hz, 5Hz) was measured using the rapid-current perception threshold (R-CPT) mode of the Neurometer (Neurotron, Incorporated, Baltimore, MD, USA) equipment. The patient was asked to stop pressing the button when he or she detected minute electrical stimulation, vibration, pain, and/or heat from electrical stimulation using the R-CPT mode. Measurements were performed repeatedly for each of the three frequencies (2KHz, 250Hz, 5Hz) until a beep was obtained from the equipment (Figure 2).

#### Thermography

Tests and treatments that stimulate the skin were prohibited before using thermography, and the patient should not apply lotions or ointments before taking images. In addition, smoking and drinking were prohibited. The images were taken after waiting for 15 to 20 minutes in the waiting room. The patient should not wear accessories such as a watch, necklaces, etc. on the image area. The room temperature should be maintained at 23- 25°C.

**Figure 3:**
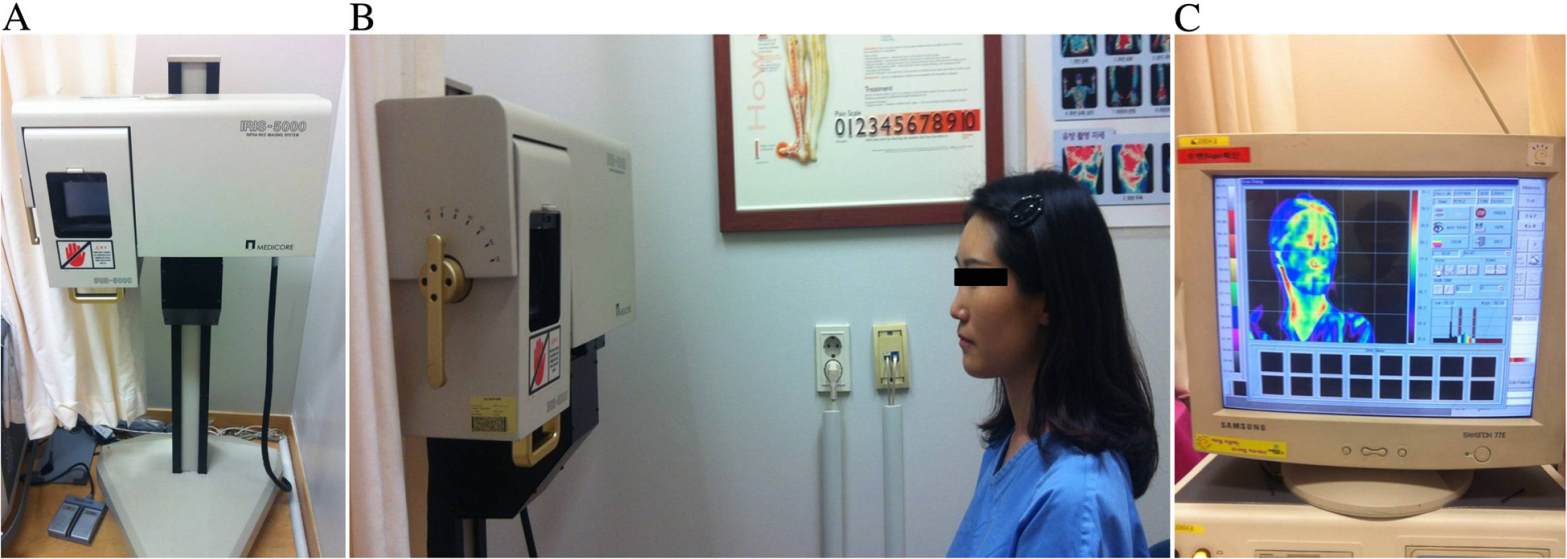
Method for taking an image with thermography. **A:** Thermography IRIS-5000. **B:** Taking the image with thermography. **C:** Temperature of the areas of interest was determined using saved images.

**Figure 4:**
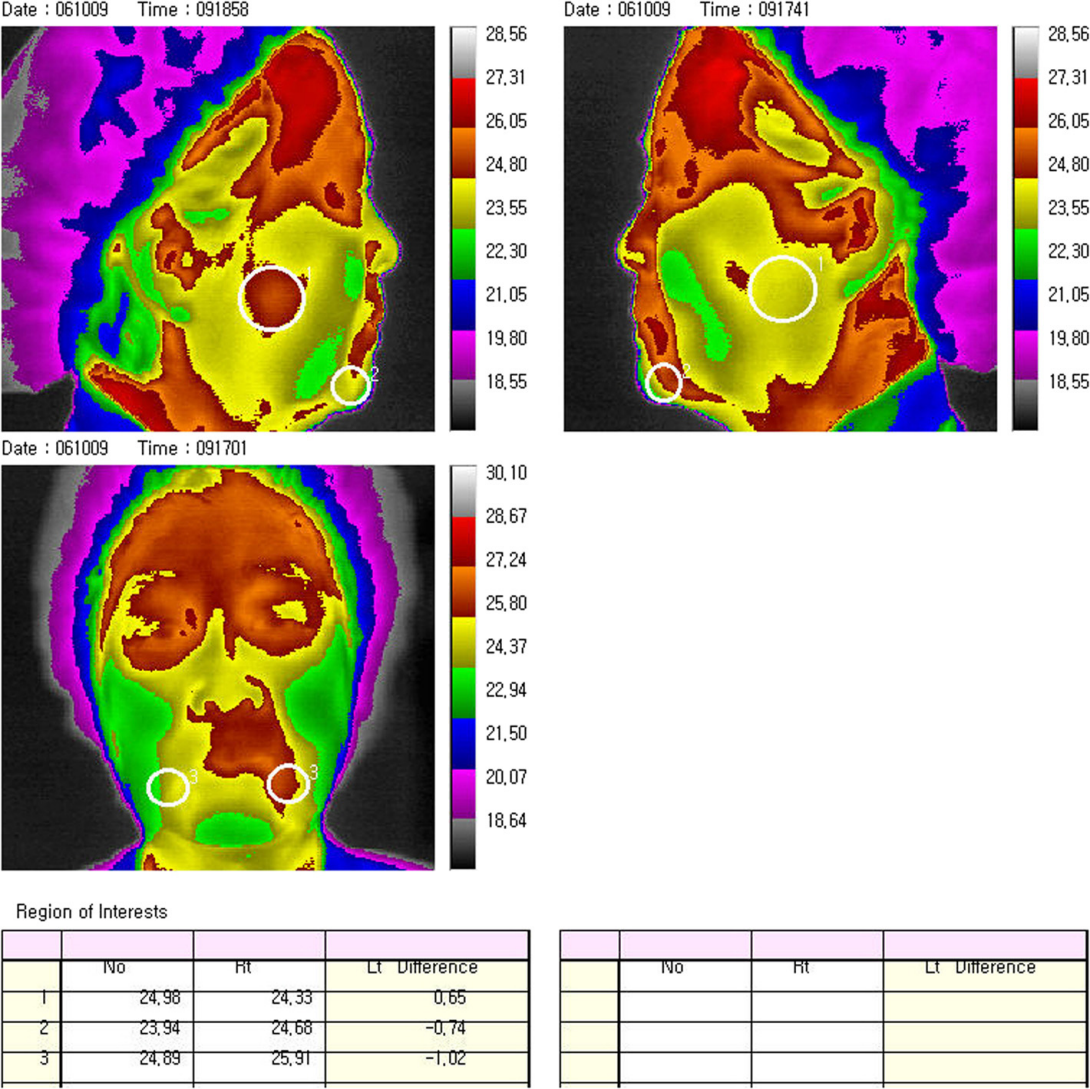
Thermography showed that the temperature differences at the region of interest No. 2(chin area) and 3(lower lip area) were 0.74 and 1.02.

Frontal and lateral images of the patient at a specific distance were taken using an IRIS 5000 (Medicore, Seongnam, Korea), and the temperature of the areas of interest was determined using saved images. Thermography involved calculation of the averages of the absolute value of the temperature difference between the abnormal and normal sides (|ΔT| = |Body surface temperature of abnormal side - Body surface temperature of the symmetric part of the normal side|) (Figure 3, 4).

#### Statistics

To compare the results of SEP and QST between the normal and abnormal sides, we used SPSS version 12.0 (SPSS Inc. Chicago, IL, USA) with the Mann–Whitney test. We also applied Wilcoxon’s signed-rank test for thermography, assuming the value for the normal side to be zero. *P* values less than 0.05 were considered to indicate statistical significance.

## Results

In the SEP test, the N20 latency of the abnormal side was 16.18 ms and that of the normal side was 15.87 ms. The abnormal side showed a more delayed pattern than the normal side; however, there were no significant differences (*P* = .163) (Table 2). In the QST tests, CPT values of the abnormal side were significantly higher at all three frequencies (Table 3). Thermography showed that the body surface temperature was higher on the normal side than the abnormal side in some patients (n = 9), but the opposite pattern was observed in others (n = 8). Overall temperature was 0.18°C higher on the abnormal side than the normal side. The mean absolute temperature difference value between the normal and abnormal sides was 0.55°C. Temperature differences between the normal and abnormal sides were not statistically significant when that of the normal side was assumed to be zero (*P* = .478) (Table 4).

**Table 2:** **Mean latency of N20 between both sides of inferior alveolar nerve area showed that the abnormal side delayed more than the normal side (p > .05)**

**Case Number**	**N20 latency(ms)**		**p-value**
	**Abnormal side**	**Normal side**	
1	16.85	16.13	
2	15.83	15.13	
3	16.5	16.35	
4	16.65	16.08	
5	16.33	16.13	
6	15.55	15.5	
7	14.85	14.25	
8	15.48	15.35	
9	16.65	16.03	
10	17.5	16.6	
11	16.08	15.2	
12	17.4	17.58	
13	16.42	16.6	
14	16.15	15.25	
15	15.28	15.25	
16	16.15	17.33	
17	15.35	15.03	
Mean	16.18	15.87	0.163

Mann–Whitney test was performed

**Table 3:** **Comparison of current perception threshold**

**Frequency**	**Abnormal side**	**Normal side**	**P-value**
2KHz	286.2 ± 209.6	142.9 ± 88.2	.002*
250Hz	156.4 ± 263.4	39.8 ± 35.4	.039*
5Hz	81.6 ± 120.8	28.9 ± 25.7	.026*

Mann–Whitney test was performed

*Indicates statistically significant difference (p < .05)

**Table 4:** **Temperature differences (TD) between both side were 0.55°C(absolute TD). P > .05**

**Case Number**	**TD(°C)**	**Absolute TD(°C)**	**p-value**
1	−0.06	0.06	
2	0.73	0.73	
3	−0.38	0.38	
4	−0.18	0.18	
5	−0.67	0.67	
6	−0.08	0.08	
7	0.18	0.18	
8	1.13	1.13	
9	1.24	1.24	
10	1.02	1.02	
11	−0.50	0.5	
12	0.19	0.19	
13	1.88	0.88	
14	−0.37	0.37	
15	0.29	0.29	
16	0.09	0.09	
17	−1.39	1.39	
Mean	0.18	0.55	0.478

## Discussion

SEP, which was developed in 1947 by Dawson, is more objective than earlier techniques and has the advantage of quantification of the extent of damage. It has been widely used since the 1960s because it is an objective, noninvasive technique and can quantify the degree of damage rapidly. Larsson and Prevec used it to evaluate the trigeminal nerve in 1970 for the first time [9]. The perceived distinction between two points was the most widely used method for damage assessment before the development of SEP [10], but it has the disadvantage of low reproducibility, with results varying depending on the patient’s general condition, emotional state, and environment. SEP, which detects the brainwave reaction induced by electrical stimulation of the peripheral sensory nerve, is an objective test for the presence of lesions and degree of somatosensory conduction in the peripheral and central nervous system [11]. It is applied clinically to evaluate the conduction of large-fiber sensory tracts in the central and peripheral nervous system, the anatomical location of the somatosensory path failure, nerve damage caused by conduction failure, and loss of sensation for nonorganic reasons. The basic forms of SEP are N and P, depending on the polarity and latency values that appear in several waveforms. The basic peak pattern that expresses sensation of the normal side in SEP of the trigeminal nerve includes the P20, N30, P40, N50, N13, P19, N26, P23, N34, N20, P34, and N51 waveforms, and the latency [9]. The peak of waveforms of normal side was observed with triphasic response. It was reported N13,P19,N26 by Badr et al. [12], N13, P19, N26 by Stohr et al. [13] and P23, N34 by Singh et al. [14] The wave patterns were N20 in this study. The amplitude and latency of SEP waveform were used for evaluation of nerve injury. Barker et al. [15] reported the severity of numbness effects to the latency of waveform in traumatic nerve injury patient. Factors that affect SEP latency include recorded region and stimulus intensity, but regardless of age, a short latency in women has been reported [15,16]. Stohr et al. reported the relevance of age and latency to the N13 waveform [13]. The N20 waveform used in this study did not show a significant delay on the abnormal side. This results from the deviation of the period from injury to treatment for each patient. Therefore, studies using a larger number of patients would likely produce more meaningful results.

QST has been used clinically in diseases of the oral and maxillofacial region, including temporomandibular joint disorders (TMD), burning mouth syndrome, malignant oral lesions, numb chin syndrome, and post-traumatic pain [17]. It is used to elucidate the mechanism of peripheral nerve function assessment and central sensitization in patients who suffer from pain [18], and has a diagnostic sensitivity of 60-85% [19]. QST is being used to evaluate the applicability of CPT to peripheral neuropathy, carpal tunnel syndrome, spinal radiculopathy, the efficacy of peripheral nerve blocks, and assessment of the hypo- and hyper-sensitivity of sensory nerves. Caissie et al. [7] reported that a significant difference in the mandibular branch of the trigeminal nerve of 50 normal subjects at 2 KHz, but no difference at 250 Hz and 5 Hz. The factors affecting CPT tests include the amount of gel applied, the position of the electrode, the attachment strength of the electrode, and the instability of 2 KHz CPT itself [20]. Yekta et al. [21] assessed trigeminal nerve functions by QST in patients and healthy volunteers. Though age, gender, and anatomic region can affect the results of the QST, they noted that the QST can be useful in the diagnosis of inferior alveolar nerve disorders. Moreover, it can be available to monitor the affected nerve for decisions about further interventions. CPT is considered a useful diagnostic method for evaluation of the damaged nerve because it was significantly higher at the injured area of the inferior alveolar nerve at 2 KHz, 250 Hz, and 5 Hz in this study.

Infrared thermography could diagnose abnormalities of the body using color images that indicate the change in body temperature resulting from pain. The amount of infrared emitted from the patient’s body is visualized in images on a monitor. This method has been applied to the diagnosis of various diseases. It was developed with the basic concept that the difference between left and right body temperature (ΔT) is in a certain range in the normal situation, but disease results in a significant temperature difference between similar body parts and body surface area. This test began was first used in the diagnosis of breast cancer patients in 1956 [22]. In dentistry, it has been used to evaluate the treatment of dental pain, endodontic experiments, and TMD, and for the assessment of inferior alveolar nerve damage [23]. Patients with inferior alveolar nerve damage have an altered skin temperature due to sympathetic vasomotor nerve damage [24].

In facial thermography studies in normal subjects, the reported temperature differences between the left and right sides (ΔT) have been less than 0.2°C; [25] in particular, the average ΔT of normal TMJ is less than 0.1°C [26]. Although extreme results in TMD patients (ΔT 0.8°C) have been reported [27], most studies have reported a ΔT = 0.40-0.43°C [28]. Thermography has potential as an auxiliary tool for assessment of the TMJ region because of its higher specificity for TMD, although its sensitivity is low [29]. At the time of diagnosis of complex regional pain syndrome, regardless of a lower or higher body temperature on the abnormal side, if its absolute value is greater than a certain level, it has significance [30]. Lee et al. [31] performed thermographic assessment of inferior alveolar nerve injury in patients with dentofacial deformity. They suggested the infrared body temperature method is an objective method that can be applied as a supplemental diagnostic method for inferior alveolar nerve injury.

In this study, the number of subjects was small and there were no statistically significant differences, but the absolute temperature difference value of 0.55 shows that this method could be used as a supplementary tool for assessment of nerve damage. The standard sensory testing methods such as 2-point discrimination threshold, temperature sensitivity, and light touch perception threshold were not used in this study because their results vary depending on the examiner’s expertise and patients’ subjective responses [32]. The patients’ subjective symptoms were very diverse, and it was difficult to classify their problems specifically, such as paresthesia, dysthesia, and anesthesia, because a significant number of patients who appeared to have anesthesia could feel pain and/or touch. We did not classify details but used the term “altered sensation” for neurologic signs and symptoms [33].

It is unclear why there were no significant differences in SEP and thermography but there was a significant difference in QST in this study. However, in patients with altered sensation, a variety of symptoms tend to appear, which are affected by a variety of nerve fibers such as thick myelinated Aß fibers for touch or proprioceptive perception, thin myelinated Aδ fibers for cold detection, and thin unmyelinated fibers for heat detection [17].

It seems that the most accurate method for investigating the response of sensory nerve fibers is QST in cases of nerve injury after dental implant placement, and SEP and thermography are ancillary diagnostic tools.

## Conclusion

These results indicate that QST is a valuable objective method for assessment of nerve injury after dental surgery. Diagnostic and prognostic decisions informed by these objective tests for nerve injury would likely be more reasonable.

## Abbreviations

SEP: Somatosensory Evoked Potentials
QST: Quantitative Sensory Testing
CPT: Current Perception Threshold
R-CPT: Rapid-current perception threshold
TMD: Temporomandibular joint disorders
